# Acquired thrombotic thrombocytopenic purpura and HIV infection: a case report and review of the literature

**DOI:** 10.1007/s00277-026-06733-7

**Published:** 2026-01-20

**Authors:** Silvio Rao, Arianna Liparoti, Juri Alessandro Giannotta, Andrea Artoni, Antonio Muscatello, Alessandra Bandera, Flora Peyvandi

**Affiliations:** 1 Angelo Bianchi Bonomi Hemophilia and Thrombosis Center, Fondazione IRCCS Ca’ Granda Ospedale Maggiore Policlinico, Milan, Italy; 2 Infectious Diseases Unit, Fondazione IRCCS Ca’ Granda Ospedale Maggiore Policlinico, Milan, Italy; 3 Department of Oncology and Hemato-Oncology, University of Milan, Milan, Italy; 4 Department of Pathophysiology and Transplantation, University of Milan, Milan, Italy

**Keywords:** Thrombotic thrombocytopenic purpura, HIV, HAART, TTP-like syndrome, ADAMTS133, Caplacizumab

## Abstract

Human immunodeficiency virus (HIV) is a rare cause of thrombotic microangiopathies (TMA), that can present either with normal ADAMTS13 activity (referred as HIV-related TMA) or suppressed ADAMTS13 activity (referred as HIV-related acquired thrombotic thrombocytopenic purpura, aTTP). The distinct characteristics and management of these two conditions is poorly known, given their rarity and often overlapping features. Here, we report the case of a 46-year-old female patient with TTP who received a diagnosis of HIV infection at her ADAMTS13 relapse and obtained complete remission only with antiretroviral therapy (ART). Moreover, we summarize the existing evidence in the literature about clinical presentation, outcomes and treatment of HIV-related aTTP/TMA.

## Introduction

Thrombotic thrombocytopenic purpura (TTP) is a rare thrombotic microangiopathy (TMA), a wide group of disorders characterized by microangiopathic hemolytic anemia (MAHA), thrombocytopenia and occlusive microvascular disease. TTP is caused by severe deficiency of ADAMTS13 (activity < 10% of normal values ), the metalloprotease normally responsible for von Willebrand factor (VWF) cleavage.

The deficiency of ADAMTS13 causes the formation of platelet-rich thrombi in the microcirculation, thus the fragmentation of red blood cells and end-organ damage predominantly affecting the brain, heart and kidneys. It represents a life-threatening medical emergency due to a sudden neurologic and cardiac dysfunction, progressing to death in 90% of cases, if not promptly treated [1].

ADAMTS13 deficiency can be congenital (~ 5% of all TTP cases), due to biallelic mutations in the ADAMTS13 gene, or acquired (~ 95% of cases, aTTP), mainly due to specific anti-ADAMTS13 autoantibodies. The latters are usually of IgG type (especially IgG1 and IgG4) or less frequently IgA or IgM. Anti-ADAMTS13 antibodies can have a neutralizing effect on the catalytic activity of the metalloprotease or a non-neutralizing action mediating the complexation of ADAMTS13 and then its clearance.

Approximately 50% of acquired thrombotic thrombocytopenic purpura (aTTP) can be secondary to viral infections (e.g. human immunodeficiency virus HIV, cytomegalovirus CMV, hepatitis B and C, COVID-19), autoimmune disorders (e.g. systemic lupus erythematosus SLE), pregnancy and, rarely, drugs like cyclosporin A or clopidogrel [2, 3].

HIV is a known cause of TMA and people living with HIV (PLWH) have an estimated risk to develop HIV-related TMA that is 15- to 40-fold higher than the general population; it is not unusual that the diagnosis of TMA leads to the detection of an unknown HIV infection.

Two main presentations have been described for the TMA secondary to HIV: some cases with severe (< 10% of normal values) ADAMTS13 deficiency (referred as HIV-related aTTP) and others with lower than normal, but still detectable ADAMTS13 activity levels (> 10% of normal values, referred as HIV-related TMA). Due to the relatively recent widespread use of ADAMTS13 testing for TTP diagnosis, the distinction between the two forms in the literature has not been always addressed, with consequently diverse treatment patterns.

Here we report the case of a patient who received a delayed diagnosis of HIV infection with severe immune deficiency at the time of ADAMTS13 relapse of TTP.

## Materials and methods

The patient’s clinical and laboratory data were collected from her medical charts, after obtaining her informed consent and in accordance with the Declaration of Helsinki.

We searched for the available evidence about the association of HIV and TTP/TMA, collecting data over the last 20 years in MEDLINE via PubMed and the National Library of Medicine (last search: October 4th, 2025).

## Results

### Case presentation

A 46-year-old Latin-American woman with no relevant medical history presented to the emergency department of another institution, with 2 months of progressive fatigue, new petechiae on limbs and gastro-enteric symptoms (vomit, constipation). Laboratory exams revealed anaemia (Hb 89 g/L), thrombocytopenia (platelets 12 × 10^9/L), mild lymphopenia (absolute lymphocyte count 1.25 × 10^9/L), slightly impaired renal function (serum creatinine 1.43 mg/dL), increased lactate dehydrogenase (1082 IU/L), total bilirubin 1.3 mg/dL, undetectable haptoglobin and schistocytes on peripheral blood smear. Coagulation parameters were normal. Given the strong suspicion of TTP, ADAMTS13 activity was promptly tested and resulted < 0.2 IU/dL with high titre of inhibitory autoantibodies (79 IU/mL), confirming the diagnosis of aTTP. Baseline serology screening performed at the local hospital (QuantiFERON-TB, HCV, HBV and HIV) were reported as negative in the clinical documentation at the time of transfer.

The patient was centralised to our intensive care unit for stabilization and subsequently admitted to our haematological division. Here, baseline imaging (total-body CT, brain MRI, mammography and breast ultrasound) and ophthalmologic check-up excluded both secondary causes and organ damage related to aTTP. Autoimmune screening tests (ANA, ENA, anti-DNA antibodies, lupus anticoagulant and anti-cardiolipin IgG/M antibodies) were unremarkable. Physical examination revealed longstanding molluscum contagiosum lesions on the groin and limbs.

At arrival, the patient started therapeutic plasma exchange (TPE; 4 once daily procedures with 3 L of plasma exchange per session), caplacizumab, and intravenous methylprednisolone 60 mg/day. She reached clinical response at day 4 (allowing TPE discontinuation) and ADAMTS13 remission (50.7 IU/dL) at day 9 [4]; caplacizumab was therefore stopped at day 17. The patient was discharged and started regular outpatient follow-up.

Two months later, she was hospitalized elsewhere for acute respiratory failure due to interstitial pneumonia. She received antibiotic course of ceftriaxone, azithromycin and oxygen-therapy supplementation with apparent clinical resolution. Isolation of Pneumocystis jirovecii DNA and CMV DNA on bronchoalveolar lavage was revealed after discharge. TTP relapse was excluded, and ADAMTS13 complete remission was confirmed during this episode.

After 2 months of wellness, she returned to our clinic with worsening fatigue. Laboratory exams revealed suppressed levels of ADAMTS13 activity (5.6 IU/dL) with still normal values of platelets and haemoglobin. Treatment with rituximab was scheduled but repeat viral screening revealed newly diagnosed HIV infection with advanced immunodeficiency (HIV-RNA 1500000 copies/mL, CD4 + lymphocyte count 135/uL (12%) and CD4/CD8 ratio 0.19 at baseline). Based on clinical suspicion of previous missed diagnosis, HIV testing was therefore performed also on retention of blood sample collected at the first admission before plasma-based treatment, and it confirmed the presence of HIV infection already at aTTP onset. Rituximab was withheld and she was hospitalized. Antiretroviral therapy (ART) with bictegravir/tenofovir alafenamide fumarate/emtricitabine was started. A brain CT scan and MRI showed a left thalamic lesion consistent with neurotoxoplasmosis. Microbiological tests on cerebrospinal fluid were negative. Treatment with pyrimethamine and sulfadiazine was started and the patient was discharged with an outpatient follow-up. About 30 days after the introduction of ART, ADAMTS13 activity raised to 106 IU/dL with no need of immunosuppressive treatments.

After only 3 weeks, she was re-hospitalized for influenza-virus B infection and acute renal failure due to sulfadiazine, that was stopped. Treatment with oseltamivir and intravenous fluid therapy was started with improvement of kidney function, as well as second-line therapy for neurotoxoplasmosis with trimethoprim/sulfamethoxazole. Follow-up brain MRI showed dimensional reduction of the thalamic lesion. Besides, cytomegalovirus reactivation occurred, thereby treatment with ganciclovir was started with benefit.

At last follow-up, 9 months after aTTP diagnosis and 3 months after the beginning of ART, the patient maintains ADAMTS13 complete remission (ADAMTS13 activity 130 IU/dL). HIV management continues successfully, with progressive viral load decline (latest: 292 copies/mL) and CD4 count improvement to 188/µL. She is currently on ART and secondary prophylaxis for neurotoxoplasmosis.

### Review of the literature

In Table 1 we summarize the pathophysiological differences between HIV-related aTTP and TMA.

**Table 1:** Main differences between HIV-related aTTP and HIV-related TMA

	HIV-related aTTP	HIV-related TMA
Occurrence in relation to the infection	Variable, but usually in patients already immune-depressed	Frequently in a more advanced stage (i.e. AIDS)
ADAMTS13 activity levels	Severe deficiency (< 10% of normal values )	Moderately low to normal (> 10% of normal values )
Grade of immunodeficiency	Moderate: T CD4 + lymphocytes > 100/uL	Severe: T CD4 + lymphocytes < 100/uL
Response to TPE	Similar to aTTP (75–96% of patients)	TPE less effective (50% of patients)

*HIV*, human immunodeficiency virus; *TTP*, thrombotic thrombocytopenic purpura; *TMA*,thrombotic microangiopathy; *AIDS*, acquired immunodeficiency syndrome; *ADAMTS13*,a disintegrin and metalloproteinase with a thrombospondin type 1 motif member 13; *TPE*, therapeutic plasma exchange

Notably, the severe deficiency of the metalloprotease found in HIV-related aTTP is often due to an acquired inhibitor (anti-ADAMTS13 autoantibodies); in the past years, some authors suggested that this was more frequent in PLWH with a CD4 + T cells > 100/uL, when autoimmune phenomena are more likely to occur [5]. However, cases in which autoantibodies are not detectable have been described, making uncertainty about their relevance in this contest [6]. Indeed, some authors suggested that the undetectability of autoantibodies is probably due to a lack of sensitivity of laboratory techniques rather than a true absence. For example, the presence of anti-ADAMTS13 IgG-ADAMTS13 antigen immune complexes might be present in vivo but undetectable in vitro, or similarly IgA and IgM type antibodies may be not detected by common antibody assays [7].

Differently, HIV-related TMA generally shows preserved ADAMTS13 activity (> 10 IU/dL), and different authors underlined a possible correlation with lower CD4 counts and a deeper immune-compromission. In such cases, TMA could be related to an important endothelial dysfunction, because of the progressive damage mediated by the HIV itself after a long-lasting infection and by the multiple opportunistic infections. A role of other immune effectors, like hyperactivation of the complement cascade, has been suggested too. This leads to a prothrombotic phenotype, due to increased release of VWF and other prothrombotic molecules from the endothelium that overcomes the cleavage capability of ADAMTS13. The prognosis of HIV-related TMA has been described as worse than that of the HIV-related aTTP [5, 6, 8].

The current standard of care for immune-mediated TTP is TPE in association with caplacizumab and immunosuppression (steroids +/- rituximab). Caplacizumab is a monoclonal, bivalent humanised antibody, that binds to the A1 domain of VWF, preventing its binding to the platelet’s GpIb-IX-V complex. Two phase 3, placebo-controlled clinical trials and several real-world evidence data have confirmed that the addition of caplacizumab to TPE warrants faster clinical responses, less exacerbations and, most importantly, reduced mortality and thromboembolic complications [9–11]. Rituximab is an anti-CD20 monoclonal antibody that has been integrated in the standard of care since 2010 as it reduces refractoriness, relapses and mortality [12].

Importantly, different studies conducted in the last two decades often used fresh frozen plasma (FFP) infusion rather than TPE in HIV-related aTTP, since the latter was not always affordable or available in all centres, reserving TPE for refractory patients. However, FFP infusion can result in the administration of insufficient amounts of plasma due to ensuing fluid overload and donors’ unavailability, together with other complications [13], thereby justifying the need to convert to TPE [6].

In patients with low ADAMTS13 levels during clinical remission, the risk of TTP relapse is 30–50%, therefore lifelong monitoring of ADAMTS13 is part of current patients’ follow-up, allowing pre-emptive rituximab treatment in case of ADAMTS13 relapse (i.e., reduction of ADAMTS13 activity levels below 20%) [14, 15].

In newly diagnosed PLWH with TTP, the main difference in the management is the concomitant highly active antiretroviral therapy (HAART), that should be initiated in parallel with TTP-specific treatments. The importance of HAART for the good control of the disease is suggested by the observation that TTP relapses typically occur in patients who discontinue the antiviral therapy, and that its prompt re-initiation along with TPE +/- steroids leads to TTP remission. Although HAART prevents TTP relapses, it is not equally efficient in preventing the first event. Indeed, some cases of TTP have been described in PLMW already on HAART, even with a controlled viral load [6]. Rituximab appears effective in PLWH with multiple TTP relapses or when TTP occurs with undetectable viral loads. Higher HIV viral loads (> 500000 copies/mL) also require more TPE to achieve remission [16].

The evidence concerning the use of caplacizumab in HIV-related aTTP is even more limited, due to its recent availability. A recent report described two PLWH who developed aTTP and were treated with caplacizumab and rituximab. Both patients achieved rapid and complete remission, with no relapses observed during the follow-up of 2 and 4 months, respectively [17].

In Table 2 we present a summary of the available evidence about the co-occurrence of TMA/TTP and HIV infection. Of note, in earlier studies plasma infusion was the first-line therapy; moreover, in some centres ADAMTS13 activity test was not routinely performed.

**Table 2:** Observational studies of patients with HIV-related aTTP

Authors, year, *N* of pts	ADAMTS13 activity	Phase of HIV infection (T CD4 + count)	Treatment	Outcome (% survival after first episode)
Louw et al. (2018) [8], 17 pts	Not available	Average CD4 count 134 (90–305/uL)	TPE + CCS + HAART	96.5%
Hart et al. (2011) [16], 24 pts	< 5% (16 pts) < 50% (8 pts)	Average CD4 count 236 (70–540/uL)	TPE + HAART +/- CCS +/- anti-CD20	96%
Gunther et al. (2007) [18], 20 pts	< 10% (14 pts) Detectable (6 pts)	Average CD4 count 236 (87–818/uL) Average CD4 count 117 (6–408/uL)	TPE/PI +/- CCS	75%
Novitsky et al. (2005) [19], 21 pts	Not available	Average CD4 count 96 (1–220/uL)	PI (TPE in non-responders) +/- CCS	95%
Masoet et al. (2017) [20], 41 pts	Not available	Average CD4 count 173 (107–261/uL)	PI (TPE in non-responders) + CCS + HAART	56%
Malak et al. (2008) [21], 29 pts	< 5% (17 pts) ≥ 5% (12 pts)	Average CD4 count 210 (30–350/uL) Average CD4 count 30 (0–620/uL)	TPE +/- CCS + HAART (+ vincristine/splenectomy in refractory/relapse or for flare-up episodes)	75% 50%
Swart et al. (2019) [22], 41 pts	Not available	Average CD4 count 137 (59–194/uL)	TPE/PI + CCS + HAART +/- Rituximab (in refractory/relapsed pts)	71% (*Remission rate 58.5%)

*PI* plasma infusion, *TPE* therapeutic plasma exchange, *CCS* systemic corticosteroids, *HAART* highly active antiretroviral therapy

## Discussion and conclusions

HIV can be associated with two forms of TMA: one with suppressed ADAMTS13 activity (HIV-related aTTP) and one with normal/moderately low ADAMTS13 activity (HIV-related TMA). Due to the different pathophysiology, the latter is less responsive to standard approaches used in aTTP, accounting for worse outcomes [5, 6, 8].

Our case represents a classical pattern of aTTP with severe ADAMTS13 deficiency rapidly responsive to TPE, steroids and caplacizumab. Apparently, the patient’s baseline microbiologic screening (including serology for HIV) performed elsewhere was negative, thus she was treated as an idiopathic form. However, the clinical presentation, characterized by concomitant opportunistic infections (neurotoxoplasmosis, CMV reactivation and *Pneumocystis jirovecii* pneumonia), better fixed with an AIDS presenter. It was only at the time of her ADAMTS13 relapse that the HIV infection was firstly detected; consequently, by repeating HIV testing on retention of blood sample stored at the moment of the first hospitalization, we clarified that the viral infection was already present when the first episode of aTTP occurred.

To the best of our knowledge, our case is the second report on the safe and successful use of caplacizumab in a patient with HIV-related aTTP. Moreover, the prompt control of HIV-related aTTP in our patient supports the well-known role of antiretroviral therapy in inducing significant immunological recovery and complete restoration of ADAMTS13 activity [8, 16, 20–22].

In conclusion, we emphasize the importance of HIV testing in all patients presenting with microangiopathic haemolytic anaemia and thrombocytopenia, as suggested by the British TTP guidelines [1], to allow a prompt recognition of HIV-related aTTP or TMA that require different treatment. The frequency of HIV re-testing in aTTP and other immune-mediated diseases is not clearcut, but this case suggests the importance of repeating viral serology before starting new treatments or even routinely in patients with risk behaviours.

## Data Availability

No datasets were generated or analysed during the current study.
